# Barshop Symposium on Aging 2017 Abstracts

**DOI:** 10.1080/20010001.2018.1444939

**Published:** 2018-03-07

**Authors:** Warren Ladiges

**Affiliations:** Editor-in-Chief

The 2017 Barshop Symposium on Aging was held at the Mayan Ranch in Bandera, Texas, Texas Hill Country, October 12-15. The theme was Sex differences in aging: Mechanisms and responses to interventions. The conference organizers were Veronica Galvan, PhD, and James Nelson, PhD from the UT Health Sciences Center at San Antonio, TX. Abstracts from the meeting are presented in this issue, and represent a variety of aging and age-related studies. A large focus was on aging and neurodegeneration including a number of presentations on Alzheimer’s disease. Other topics included cardiomyopathy, frailty, metabolism, pharmaceutical intervention of aging, age-related epigenetic modification, and cancer and aging. This annual meeting is well-attended and provides a seclusive and rustic environment in the Texas Hill Country for small group presentations and extensive informal interactions and discussions on the very latest findings and current thinking in the causes and prevention of aging and age-related diseases.

## LDLR-related protein 1 increases cytokine sensitivity. Implications for recovery after brain damage

Sadiya Ahmad^1,*^, Pamela Reed^1^, Shane Sprague^1^, Naomi L. Sayre^1^

^1^University of Texas Health Science Center at San Antonio (UTHSCSA), TX

Patients that express the Apolipoprotein E4 are predisposed to a poor long-term outcome after stroke. Explanations for this increased risk are not yet elucidated. This study aims to test one possible mechanism by which ApoE4 contributes to cognitive decline after stroke. Here, we examine the effect of a major ApoE4 receptor, low-density lipoprotein receptor related protein 1 (LRP1) on sensitivity to stress in astrocytes. LRP1 can promiscuously bind and move several extracellular ligands and plasma membrane proteins into the endocytic system. Notably, LRP1 was previously found to remove the TNF receptor (TNFR1) from the plasma membrane, although this has not been shown in astrocytes. We propose that a similar mechanism occurs in the central nervous system to attenuate inflammatory response after stroke. LRP1 binds and clears ApoE4 from the extracellular space via receptor-mediated endocytosis. However, previous studies have shown that the ApoE4, compared to other ApoE isoforms, slows the trafficking and recycling of endocytic LDL receptors. We propose that ApoE4 similarly inhibits LRP1 trafficking, and hypothesize that ApoE4 inhibits the ability of LRP1 to remove TNFR1 from the plasma membrane. This is expected to increase cytokine sensitivity, which would result in worse outcome after stroke and with aging. We investigated the effect of LRP1 on astrocyte TNFα signaling and response in immortalized ApoE null mouse astrocytes subjected to lentiviral-mediated knockdown ofLRP1. The astrocyte response to TNFα stimulation was tested in a concentration dependent manner using Western blotting of NFkB pathway components, which are the downstream mediators of TNFα signaling. We also tested astrocyte viability after prolonged TNFα stimulation using Alamar Blue reagent. We found that LRP1 deficient cells have increased phosphorylation of NFkB upon TNFα stimulation, and that loss of LRP1 resulted in significant loss of astrocyte viability after prolonged stimulation. Altogether, our results indicate that loss of LRP1 renders astrocytes more sensitive to TNFα. Future experiments will focus on treating astrocytes ApoE4 to determine if detrimental effects are exerted through LRP1, as well as testing the influence of LRP1 on recovery after middle cerebral artery occlusion in mice.

*Correspondence to: Sadiya Ahmad, UTHSCSA, TX, USA. Email: ahmedsn@uthscsa.edu

## Elucidating the role of ALCAT-1 in dilated cardiomyopathy

John-Paul Andersen^1,*^

^1^Barshop Institute, UTHSCSA, TX

Dilated cardiomyopathy (DCM) is a disease characterized by an abnormally large and weakened left ventricle which impairs the heart’s ability to pump blood. Due to its high mortality rate and high prevalence, DCM has been extensively researched and studied with a variety of treatment and symptom management options available. However, despite the many advances in the field, most cases are classified as idiopathic and most individuals die within two years of diagnosis. What has yet to be elucidated as a possible contributor to DCM is pathological cardiolipin remodeling. A major indicator that points to pathological cardiolipin remodeling being a primary contributor to DCM is the rare, x-linked genetic disorder Barth Syndrome. In this disease, individuals have a mutated tafazzin gene which causes a decrease in the predominant healthy species of cardiolipin known as tetralinoleoyl cardiolipin. Tetralinoleoyl cardiolipin has been shown to be drastically decreased in rodent models of heart disease as well as in humans with cardiomyopathy. Through investigating the role of cardiolipin remodeling on DCM and altering which cardiolipin species are present, it may open up novel treatment options for the disease and help provide greater insight into the cause of DCM.

*Correspondence to: John-Paul Andersen, UTHSCSA, TX, USA. Email: andersenj@uthscsa.edu

## Evaluation of Long-Term Hippocampal NF-κB Suppression on Murine Behavior and Tau Protein Expression

Eric Baeuerle^1,*^, Miranda Orr^1^, Ning Zhang^1^, Joseph M. Valentine^1^, Hanyu Liang^1^, You Zhou^1^, Nicolas Musi^1^

^1^Barshop Institute, UTHSCSA, TX

Tau proteins are most well known for their role in neurodegenerative diseases such as Alzheimer’s disease. Physiologic tau regulates and stabilize microtubules within neuronal axons. Abnormal tau hyper-phosphorylation leads to disruption of microtubule stabilization and subsequent tau aggregation, which can lead to neurodegeneration and dementia seen in Alzheimer’s disease. Although inflammation is known to be a major contributor to Alzheimer’s disease progression, its association with tau protein expression and regulation has not been significantly examined. Prior work by our research group has shown that inhibition of the transcription factor NF-ĸB, which directly regulates the expression of numerous inflammatory cytokines, increases tau protein expression in neuronal cells. In our ongoing studies, we are examining whether NF-ĸB regulates tau *in vivo* using stereotaxic delivery of AAV-IĸBα^DN^ super repressor or AAV-GFP targeted to young mouse hippocampal neurons. After long-term suppression of hippocampal NF-ĸB, cognition and behavior were evaluated in a blinded manner using assays including open field, novel object recognition, Morris water maze, and contextual fear. Significant differences were observed between NF-ĸB suppressed and control groups in behavioral analysis. Whole brain and hippocampal tissue were collected for immunofluorescence, protein and RNA evaluation of NF-ĸB suppression, tau protein expression and regulation, and other associated markers of neuro inflammation. Future experiments will investigate hippocampal-specific upregulation of NF-ĸB-associated inflammation. The results of this work will help elucidate novel mechanisms of tau protein regulation and provide understanding of the role of inflammation and tau in the initial pathogenesis of Alzheimer’s disease.

*Correspondence to: Eric Baeuerle, UTHSCSA, TX, USA. Email: baeuerle@livemail.uthscsa.edu

## Previous midlife estradiol treatment results in increased nuclear erα expression in the hippocampus of aging ovariectomized rats

Nina E. Baumgartner^1,2,*^, Katelyn L. Black^1,2^, Jill M. Daniel^1,2,3^

^1^Neuroscience Program; ^2^Brain Institute; ^3^Department of Psychology Tulane University

Work from our lab has demonstrated that previous midlife estradiol treatment improves memory in ovariectomized female rats months after hormone exposure has ended. Furthermore, midlife estradiol exposure results in lasting increases in levels of estrogen receptor alpha (ERα) in the hippocampus, an effect that mediates the memory enhancements. Traditionally, ERα acts as a nuclear receptor, initiating genomic effects including increased transcription of certain genes. More recently, ERs have been localized to the membrane. Activation of membrane ERα could result in non-genomic, rapid acting effects. The goal of the current work is to determine where ERα is localized following midlife estradiol treatment. Middle-aged rats were ovariectomized and implanted with hormone capsules containing either estradiol or vehicle. Forty days later, capsules were removed. One month after hormone treatment ended, rats were killed and hippocampi were dissected and processed for subcellular fractionation. Hippocampal lysate was homogenized and separated into cytosolic, membrane, and nuclear compartments using the protocol included with a commercially available kit. All samples (cytosolic, membrane, and nuclear) were further processed for western blotting for ERα. Previous estradiol treatment resulted in lasting increases of nuclear protein expression of ERα compared to vehicle-treated rats. There were only trace amounts of cytosolic ERα, regardless of hormone treatment. We found no differences in membrane ERα protein expression. Results demonstrate that in the aging female hippocampus, lasting increases in ERα protein expression following midlife estradiol treatment are due to increases in nuclear-localized ERα.

This work was supported by *National Institute of Aging* Grant RO1AG041374

*Correspondence to: Nina E. Baumgartner, Neuroscience Program, Tulane University, Louisiana, USA. Email: nbaumgar@tulane.edu

## Investigating loss of terminal neuronal differentiation in Alzheimer’s disease and related tauopathies

Adrian Beckmann^1,*^, Maria Gamez^2^, Wenyan Sun^2^, and Bess Frost^2^

^1^Institute of Biotechnology, ^2^Department of Cell Systems and Anatomy, Barshop Institute, UTHSCSA, TX

Tauopathies are progressive neurodegenerative disorders that are defined histologically by deposits of insoluble, hyperphosphorylated tau protein in the brain. Alzheimer’s disease is the most common tauopathy, with reported cases approaching nearly 50 million, globally. Current FDA-approved treatments delay the breakdown of acetylcholine with modest, temporary improvements in cognitive function. Alternative strategies target amyloid-beta (Aß) and neuro-inflammation, but have yet to arrest, alter, or reverse the neurodegenerative process. Recently, tau has manifested as a promising avenue for therapeutic intervention for many reasons. First, pathological tau correlates more closely with cognitive dysfunction than Aß in Alzheimer’s disease. Second, deposits of Aß plaques in an Alzheimer’s disease mouse model fail to induce neuronal loss in the absence of tau. Third, multiple mouse models demonstrate that tau acts downstream of Aß to mediate neurotoxicity. Recently, our laboratory has reported pathological tau can over stabilize filamentous actin, which disrupts the lamin nucleoskeleton. Further, nucleoskeletal disruptions lead to relaxation of heterochromatin, which can drive abnormal cell cycle entry and gene expression. Preliminary data suggest that transcription factors that maintain a terminally differentiated state are downregulated in adult tau-transgenic Drosophila. Thus far we have identified three critical terminal selectors known for promoting neuronal differentiation such as prospero, midlife crisis, and nerfin-1 that are differentially expressed in our Drosophila model. We are currently testing the hypothesis that neurons harboring pathological tau fail to maintain a terminally differentiated state and lead to neuronal death. If correct, therapeutic strategies aimed towards maintaining terminal differentiation in neurons may ameliorate tau-induced neurotoxicity.

*Correspondence to: Adrian Beckmann, Institute of Biotechnology, Barshop Institute, UTHSCSA, TX. Email: beckmann@livemail.uthscsa.edu

## The inverse relationship between body weight and longevity is stronger in males and weakens temporally until it reverses in senescence: Results from a large multi-site study in genetically heterogeneous mice

Catherine J Cheng^1,*^, Jonathan A L Gelfond^1^, James F Nelson^1^

^1^University of Texas Health Science Center at San Antonio (UTHSCSA), TX

The female survival advantage is one of the most robust characteristics of human longevity, but the underlying biological mechanisms are unknown. The National Institute on Aging’s Interventions Testing Program (ITP) was designed to overcome the limitations of inbred strains by evaluating lifespan-extending compounds in genetically heterogeneous HET3mice. Our detailed analysis of the survival characteristics of ~1700 mice of each sex in this population has revealed a female survival advantage that parallels that of human populations. The female survival advantage is greatest in early adulthood and diminishes progressively thereafter. This finding is observed at the 3 ITP study sites and in 6 separate cohorts over a10-yr span. Here we explore the potential role of the commonly observed inverse relationship between body weight and lifespan in the sexual dimorphism in survival of HET3 mice. The inverse relationship between body weight and longevity, although present in both sexes, is much stronger in males. While the heavier weights of males accounts for at least part of their increased mortality, the effect of bodyweight on lifespan is larger in males than in females. At 24 months, nearing the median lifespan, the relationship between body weight and longevity shifts from negative to positive in both sexes, similar to the human condition.

Supported by a Glenn predoctoral fellowship & a NIA training fellowship to CJC, and a grant from the NIA.

*Correspondence to: Catherine J Cheng, UTHSCSA, TX. Email: ChengCJ@livemail.uthscsa.edu

## The adapted endoplasmic reticulum (ER)-associated protein quality control (ERQC) is critical for the long-lived *Caenorhabditis elegans rpn-10* proteasome mutant

Meghna N. Chinchankar ^1,2,3,*^, Sarah K. Maddux ^1,2,3^, Karl A. Rodriguez ^1,4^, Alfred L. Fisher ^1,2,3,5^

^1^Barshop Institute, ^2^Center for Healthy Aging, School of Medicine, ^3^Division of Geriatrics, Gerontology, and Palliative Medicine, Department of Medicine, ^4^Department of Cell Systems and Anatomy, UTHSCSA, San Antonio, TX, ^5^San Antonio GRECC, South Texas VA Healthcare System, San Antonio, TX

The ubiquitin-proteasome system (UPS) protein degradation mechanism is integral for the optimal preservation of the proteome. Its reduced efficiency during aging results in protein misfolded and aggregation which potentiate several proteotoxic disorders. Paradoxically, our lab reported that the *Caenorhabditis elegans* proteasomal *rpn-10(ok1865)* mutant exhibits enhanced proteostasis and extended longevity. The RPN-10/PSMD4 subunit is a 19S regulatory particle (RP) ubiquitin receptor of the 26S proteasome that targets polyubiquitinated substrates to the 20S core for degradation. The *rpn-10* mutant, which shows reduced proteasome-mediated ubiquitin-fusion degradation (UFD), is remarkably resistant to many proteomic challenges. While this may be attributed to its distinct proteasomal proteolysis activity, we also sought to elucidate its suite of protective mechanisms. Analyzing our RNA-sequencing data and genome-wide RNAi screen for UFD players, we found that several endoplasmic reticulum (ER) protein quality control (ERQC) genes were upregulated in the *rpn-10* mutant. Therefore, we hypothesized that the *rpn10* mutant might exhibit enhanced ERQC or ER-associated protein degradation (ERAD). Indeed, I found that the *rpn-10* mutant cytoplasmic proteostasis and longevity benefits are highly contingent on the ER master chaperone *hsp-3/-4* (BiP/grp78) and ERAD ATPase *cdc-48.2* (p97/VCP/CDC48). Additionally, the attenuated accumulation of the aggregation-prone ER substrate α-1 antitrypsin proves that ER proteostasis is ameliorated in the *rpn-10* mutant. Moreover, the *rpn-10* mutant exhibits higher ER stress resistance but lower ER stress induction than the wild-type, which suggests its optimized ER homeostasis. Therefore, I postulate that the *rpn-10* mutant possesses adapted ERQC which critically links graded proteasomal function with improved proteostasis and increased lifespan.

*Correspondence to: Meghna N. Chinchankar, Barshop Institute, UTHSCSA, TX. Email: Chinchankar@livemail.uthscsa.edu

## Consequences of nucleoplasmic reticulum expansion in tauopathy: a possible role for aberrant nuclear RNA export in tau pathogenesis

Garrett Lee Cornelison^2,*^ and Bess Frost^1,2^

^1^Barshop Institute, ^2^Department of Cell Systems and Anatomy, UTHSCSA, TX

Laminopathies are a group of rare hereditary degenerative disorders characterized by aberrant nuclear architecture and genetic dysregulation that lead to progeroid or “advanced aging” phenotypes. Using a *Drosophila melanogaster* model of tauopathy and postmortem brain tissue from patients with Alzheimer’s disease, we have recently established a novel mechanism of acquired neuronal lamin dysfunction by which pathological tau triggers the formation of invaginations in the nuclear lamina of neurons. Interestingly, increased nuclear invagination has also been reported to occur with normal physiological aging, suggesting that exacerbation of this process by tau may underlie the association between aging and an increased risk of developing Alzheimer’s disease and related tauopathies. These invaginations, referred to as the “nucleoplasmic reticulum,” are thought to bring functions of the nuclear periphery, such as the nucleocytoplasmic transport of macromolecules, into the nuclear interior. We have found that the nuclear invaginations observed in patients with Alzheimer’s disease are lined with nuclear pores and previous studies have reported that nuclear invaginations often terminate at nucleoli, areas of high transcriptional activity. Based on these observations, together with our previous findings of heterochromatin relaxation in tauopathies, we hypothesize that increased transcription and nucleocytoplasmic export of RNA through these invaginations may underlie tau-mediated neuronal death. Preliminary studies support this hypothesis, as both genetic knockdown and pharmacological inhibition of nuclear RNA-export machinery ameliorate tau-induced neurodegeneration *in vivo*. Additionally, FISH/IF experiments have revealed a strong association of poly(A) RNAs co-localized with lamin invaginations in a *Drosophila* model of tauopathy.

*Correspondence to: Garrett Lee Cornelison, Department of Cell Systems and Anatomy, UTHSCSA, TX. Email: cornelisong@uthscsa.edu

## Impact of pre-pubertal and adult gonadectomy on sex differences in impulsive behavior in adult rats

J. S. Darling^1,2,*^, A. Mehrotra^1,2^, W. L. Smith^1,2^, J. M. Daniel^1,2,3^

^1^Tulane Neuroscience Program, ^2^Tulane Brain Institute, ^3^Tulane Psychology Department, Tulane University, Louisiana

The goal of the current work was to examine the influence of pubertal and adult gonadal hormones on sex differences on measures of impulsivity. Three sets of male and female rats were used in the study. The first set was gonadectomized prior to puberty (at 28 d of age), the second set was gonadectomized in adulthood (at 90 d of age), and the third set underwent sham surgeries and served as gonadally intact controls. Beginning at ~100 d of age, all rats were trained on the 5-choice serial-reaction time task (5-CSRTT), a test for impulsive action. The task requires rats to identify (via nose poke) the location of a brief light stimulus among five possible locations. When training was completed, impulsive action, as measured by premature responding, was assessed during sessions in which the onset of the stimulus was unpredictably lengthened. After testing on the 5-CSRTT was completed, rats were trained on a delayed-based reward task that measures impulsive choice, demonstrated as aversion to delayed reward. The task requires rats to choose between a delayed large food reward and an immediate small food reward. On the 5CSRTT, adult males made significantly more impulsive (i.e. premature) responses across all hormone treatment conditions indicating that neither gonadal hormone actions during puberty nor during adulthood mediates the observed sex difference in impulsive action. On the delay-based reward task, no sex difference in impulsive choice was observed across all hormone treatment conditions. Future studies will examine neonatal organizational effects of gonadal hormone exposure on adult sex differences in impulsive action.

*Correspondence to: Jeffery. S. Darling, Tulane Neuroscience Program, Tulane University, Louisiana. Email: jdarling@tulane.edu

## Second X chromosome decreases motor impairments in aging mice

Emily Davis^1,*^, Arturo J. Moreno^1^, Arthur P. Arnold^2^, Dan Wang^1^, Dena B. Dubal^1^

^1^Department of Neurology, Weill Institute for Neurosciences, Biomedical Sciences Graduate Program, UCSF, CA ^2^Department of Integrative Biology and Physiology, UCLA, CA

Motor impairment is emblematic of frailty in aging. To combat this biomedical problem, it is crucial that we understand major factors involved. Sex is one such factor – females (XX) live longer than males (XY) and have reduced frailty in aging. We investigated the role of sex chromosomes in aging and frailty using XY* model mice. Progeny of XY* males crossed with XX females include four sex genotypes roughly equivalent to: XX and XO mice with ovaries, and XY and XXY mice with testes. A sexual dimorphism that varies by the presence or absence of a Y is Y-chromosome-mediated; one that varies by the presence of 1 versus 2 X’s, is X chromosome-mediated. We gonadectomized mice to decrease activational effects of gonadal hormones. We then assessed motor learning, memory, coordination, and strength using the rotarod task and grip measures. We found that all genotypes of aging mice were impaired across measures compared to young mice. The presence of a second X chromosome improved motor learning and memory in aging males and females – without altering grip strength or maximal motor performance. This indicates that the second X chromosome probably enhances neural mechanisms of motor learning, memory and coordination. Effects of the second X chromosome extended to young mice, suggesting that it may confer ‘functional reserve’ to counter impairments in aging. Our results indicate a role for the second X chromosome in countering effects of aging. Understanding how an additional X confers neural resilience could lead to novel therapies for women and men.

*Correspondence to: Emily Davis, Department of Neurology, Weill Institute for Neurosciences, Biomedical Sciences Graduate Program, UCSF, CA. Email: emily.davis3@ucsf.edu

## β-Guanidinopropionic acid as an intervention targeting health-span

Dorigatti JD^1,*^, Liu Y^1^, Thyne KM^1^, and Salmon AB^1^

^1^Barshop Institute, UTHSCSA, TX

The AMP-Activated Protein Kinase (AMPK) signaling pathway plays a key role in lifespan and health-span. In *Drosophila*, genetic manipulation of AMPK expression has been shown to extend lifespan. Similarly, the anti-hyperglycemic drug metformin, an AMPK activator, has been reported to increase lifespan in mice. β-Guanidinopropionic acid (β-GPA) is a naturally occurring compound similar to creatine that acts as a competitive inhibitor of creatine phosphokinase, and is thought to activate AMPK signaling by reducing the effective ATP concentration in the cell. Based on previous studies, we investigate both the mechanism and efficacy of β-GPA as a potential intervention to improve health-span across models of aging. In the first model, *Drosophila* are treated chronically with β-GPA starting at a young age to test whether this drug delays age-related declines in voluntary motility or intestinal barrier function. Using the powerful genetics of this model, we will also identify whether the effects of β-GPA require AMPK signaling and what specific downstream mediators are involved. In the second model, we test whether late-life intervention with β–GPA can delay declines in health span in genetically heterogeneous mice. HET3 mice at 20 months of age are treated with β-GPA in the diet. In contrast to previous studies in young rodents, we report that intervention with 1% β-GPA protects both lean and fat mass in males but not females when delivered late in life. Together these models will allow us to more precisely define the mechanism and effectiveness of β-GPA in mediating health-span in multiple aging models.

*Correspondence to: Jonathan Dorigatti, Barshop Institute, UTHSCSA, TX. Email: dorigatti@uthscsa.edu

## Early and sustained microglia activation contributes to age-associated reductions in neurogenesis

Rene Solano Fonseca^1,*^, Swetha Mahesula^1^, Deana M Apple^1^, Allison Dugan^1^, Astrid Cardona^2^, Jason O’Connor^1^, Erzsebet Kokovay^1^.

^1^The University of Texas Health Science Center at San Antonio, ^2^The University of Texas at San Antonio.

The ventricular-subventricular zone (V-SVZ) is the largest neural stem cell (NSC) reservoir of the mammalian forebrain. However, NSC proliferation and neurogenesis is sharply reduced at mid-age through unknown mechanisms. Our studies establish microglia, the resident immune cells in the brain, as integral V-SVZ niche cells closely associated with NSCs, germinal pinwheels and the microvasculature. During aging, microglia undergo substantial positional changes within the niche, losing their close association to the vasculature while becoming increasingly associated with the ependyma and germinal pinwheels. We observed an early and chronic activation of V-SVZ microglia not seen in microglia outside of the niche during aging. This activation was accompanied by increased inflammatory mediators within the NSC compartment. A substantial increase of monocyte infiltration was observed within the aged V-SVZ niche, suggesting the peripheral immune system may also mediate V-SVZ inflammation during aging. Induction of sustained inflammation in young mice results in increased microglia activation accompanied by reduced proliferation in the V-SVZ and in vitro studies revealed secreted factors from activated microglia reduced proliferation and neuron production compared to secreted factors from resting microglia. Furthermore, minocycline treatment in aged mice reduces microglia activation, niche inflammation and partially restores proliferation in the aged niche. Interestingly, microglia depletion in the young V-SVZ results in a reduction of proliferation that is restored after microglia numbers are allowed to normalize. Our results suggest that age-associated chronic inflammation contributes to declines in NSC function within the aging neurogenic niche and microglia may sustain or negatively affect neurogenesis depending on age.

*Correspondence to: Rene Solano Fonseca, UTHSCSA, TX. Email: SolanoFonsec@livemail.uthscsa.edu

## Sexually divergent DNA methylation patterns with hippocampal aging

Dustin R. Masser^1,2,11^, Niran Hadad^1,3,11^, Hunter L. Porter^1,3,11^, Colleen A. Mangold^4^, Archana Unnikrishnan^1,10,11^, Matthew M. Ford^5^, Cory B. Giles^6^, Constantin Georgescu^6^, Mikhail G. Dozmorov^7^, Jonathan D. Wren ^6,8,11^, Arlan Richardson^1,2,9,10,11^, David R. Stanford^1,2,11^, Willard M. Freeman^1,2,3,10,11*^

^1^Reynolds Oklahoma Center on Aging, ^2^Department of Physiology, ^3^Oklahoma Center for Neuroscience, University of Oklahoma Health Sciences Center, ^4^Department of Biochemistry and Molecular Biology, Pennsylvania State University, ^5^Division of Neuroscience, Oregon National Primate Research Center, Beaverton, Oregon, ^6^Arthritis & Clinical Immunology Program, Oklahoma Medical Research Foundation, ^7^Department of Biostatistics, Virginia Commonwealth University School of Medicine, ^8^Department of Biochemistry and Molecular Biology, University of Oklahoma Health Sciences Center, ^9^Oklahoma City VA Medical Center, ^10^Department of Geriatric Medicine, University of Oklahoma Health Sciences Center, ^11^Oklahoma Nathan Shock Center for Aging, Oklahoma City, OK

DNA methylation is a central regulator of genome function and altered methylation patterns are indicative of biological aging and mortality. Age-related cellular, biochemical, and molecular changes in the hippocampus lead to cognitive impairments and greater vulnerability to neurodegenerative disease that varies between the sexes. The role of hippocampal epigenomic changes with aging in these processes is unknown as no genome-wide analyses of age-related methylation changes have considered the factor of sex in a controlled animal model. High-depth, genome-wide bisulfite sequencing of young (3 month) and old (24 month) male and female mouse hippocampus revealed that while total genomic methylation amounts did not change with aging, specific sites in CG and non-CG (CH) contexts demonstrated age-related increases or decreases in methylation that were predominantly sexually divergent. Differential methylation with age for both CG and CH sites was enriched in intergenic and intronic regions and underrepresented in promoters, CG islands and specific enhancer regions in both sexes suggesting that certain genomic elements are especially labile with aging, even if the exact genomic loci altered are predominantly sex-specific. Life-long sex differences in autosomal methylation at CG and CH sites were also observed. The lack of genome-wide hypomethylation, sexually divergent aging response, and autosomal sex differences at CG sites were confirmed in human data. These data reveal sex as a previously unappreciated central factor of hippocampal epigenomic changes with aging. In total, these data demonstrate an intricate regulation of DNA methylation with aging by sex, cytosine context, genomic location, and methylation level.

*Correspondence to: Willard M. Freeman, Oklahoma Nathan Shock Center for Aging, Oklahoma City, OK. Email: wfreeman@ouhsc.edu

## Developmental prozac exposure sex-dependently reduces preference for sociability in female C57BL/6J mice

Valentina R. Garbarino^1,*^, Anastassia Nelson^1^, Taylor Santos^1^, Martin A. Javors^2^, Lynette C. Daws^1^, Georgianna G. Gould^1^

^1^Cellular and Integrative Physiology Department, UTHSCSA, ^2^Physicaitry Department, School of Medicine, UTHSCSA

Autism is a developmental disorder that affects an alarming number of individuals (1 in 68 children in the US). Autism is persistent, so it impairs an individual throughout their lifespan, and often requires antipsychotic treatments, which increase incidence of age related diseases such as obesity, diabetes and heart disease with long-term use. This cycle imposes a great financial burden, as intensive health care is needed earlier in individuals with autism than in the general population. Our research goal is to reduce autism incidence by studying a potential environmental etiology of autism: developmental exposure to a selective serotonin reuptake inhibitor (SSRI) such as Prozac (fluoxetine). Our research hypothesis is that exposure to maternally administered fluoxetine (10 mg/kg/day) through gestation and lactation will increase autism-like behaviors in mice. Because serotonin plays many critical roles in early brain development, we also hypothesized that a dietary supplementation of the essential amino acid tryptophan(TRP) could prevent social and repetitive behavioral deficits from occurring in fluoxetine exposed offspring. So far, we have found female offspring from dams receiving this clinically relevant dose of Prozac throughout pregnancy and lactation displayed decreased social interaction preference, which was not rescued by the tryptophan supplement. Pilot measures of offspring serotonin and its metabolite 5hydroxyindoleacetic acid show there may be reduced serotonin turnover in the brains of fluoxetine-exposed offspring. Also fluoxetine treated dams took longer to get pregnant, and weaned fewer pups than vehicle controls. This work lays the foundation for understanding how fluoxetine may be an autism risk factor.

*Correspondence to: Valentina R. Garbarino, Cellular and Integrative Physiology Department, UTHSCSA. Email: garbarino@livemail.uthscsa.edu

## Sex-differences in lifespan-extension with acarbose and 17-α estradiol: gonadal hormones underlie male-specific improvements in glucose tolerance, motor function and muscle aging

Michael Garratt^1*^, Brian Bower^1^, Gonzalo G. Garcia^1^, Richard A. Miller^1,2^

^1^Department of Pathology, University of Michigan Medical School, Ann Arbor, MI. ^2^University of Michigan Geriatrics Center, Ann Arbor, MI

Interventions that extend lifespan in mice can show substantial sexual dimorphism. We find that male-specific lifespan extension with two pharmacological treatments, acarbose (ACA) and 17-α estradiol (17aE2), is associated, in males only, with a range of metabolic benefits in adulthood and anti-aging effects on neuromuscular and cardiovascular function later in life. Females, which show either smaller (ACA) or no lifespan extension (17aE2), do not derive these benefits from drug treatment. We find that male-specific metabolic improvements in adulthood are associated with enhanced hepatic mTORC2 signaling and increased Akt activity – changes that might promote metabolic health and survival in males. By manipulating sex-hormone levels through gonadectomy, we show that sex-specific changes in these metabolic pathways are modulated, in opposite directions, by both male and female gonadal hormones. Male castration, in particular, inhibits males from showing metabolic improvements with either drug treatment. Castrated males also do not gain the anti-aging benefits for neuromuscular function that these drugs provide in intact males. Our results demonstrate that drugs generating sex-specific lifespan extension can also improve mouse metabolism and slow age-related declines in functional traits in a sex-specific way. These sex-specific effects are influenced by the presence of gonadal hormones produced in adulthood, suggesting that gonadally-derived hormones contribute to sexual dimorphism in response to interventions that extend mouse lifespan.

*Correspondence to: Michael Garratt, Department of Pathology, University of Michigan Medical School, Ann Arbor, MI. Email: garrattm@med.umich.edu

## HP1γ in sexual dimorphism and telomere maintenance

Audrey Goldfarb^1,2,5,*^, Pui Pik Law^1,2,3^, Jean Baptiste Vannier^1,4^, Rosa Maria Porreca^1,4^, Jackson Chan^1,2^, Richard Festenstein^1,2^

^1^Gene Control Mechanisms and Disease Group, Department of Medicine, Imperial College London, London, United Kingdom ^2^Epigenetics Section, MRC London Institute of Medical Sciences, Faculty of Medicine, Imperial College London, London, United Kingdom ^3^Department of Pediatrics & Adolescent Medicine, LKS Faculty of Medicine ^4^MRC London Institute of Medical Sciences, Faculty of Medicine, Institute of Clinical Sciences, Imperial College London, London United Kingdom ^5^Biology Department, University of Rochester, Rochester, New York.

Epigenetic modifications of chromatin play many roles in chromatin structure and gene expression. It has been suggested that abnormalities in the epigenetic code might lead to sexual dimorphic diseases. Heterochromatin protein 1 (HP1)-alpha, beta and gamma are epigenetic modifiers of chromatin important for regulating gene expression, centromere stability, and telomere capping. Unlike the other two HP1 isoforms, HP1 gamma is present in both euchromain and heterochromatin, and previous data shows that it plays a role in both the repression and expression of genes in a sexually dimorphic fashion. We have preliminary data suggesting that HP1 gamma binds preferentially to female telomeres. In this preliminary study, wild type and HP1γ knock-out primary mouse embryo fibroblasts were assayed for telomere instability, fusion and loss. Our results obtained from HP1γ -/-female cells showed significantly higher telomere instability compared with cells from wildtype littermates. These results implicate HP1γ in preventing telomere fragility, which, due to its preferential binding to female telomeres, may provide a novel mechanism for sexual dimorphism.

*Correspondence to: Audrey Goldfarb, Gene Control Mechanisms and Disease Group, Department of Medicine, Imperial College London, London, United Kingdom, Email: agoldfa5@u.rochester.edu

## Reversing age-related decline using non-cytotoxic transplantation of young hematopoietic stem cells

Michael Guderyon

University of Texas Health Science Center at San Antonio

Aging of the hematopoietic system is associated with various blood disorders in the elderly. Treatment options for these patients are limited to transplantation of compatible donor hematopoietic stem cells (HSCs). Despite over 40 years of progress and innovation, hematopoietic stem cell transplantation (HSCT) is reserved for patients diagnosed with life-threatening diseases due to cytotoxic pre-conditioning such as irradiation or chemotherapy and graft versus host complications. The long term objectives for the proposed research is to develop a noncytotoxic stem cell therapy tailored to reverse aging, with the ultimate goal of treating humans. Rejuvenation of aged HSCs has been achieved through pharmacological, genetic manipulative, and caloric restrictive methods. However, permanent damage to bone architecture from cytotoxic transplantation limits health-associated benefits from rejuvenated HSCs. In this application, we will employ our novel (patent pending) transplantation regime, free from the deleterious effects of current HSCT methods, to investigate the health-associated benefits of transplanting young HSCs into aged recipients. During preliminary studies, we have achieved 90% donor engraftment and a 17% increase in median lifespan in aged recipients compared to non-transplanted controls using our non-cytotoxic pre-conditioning regime. Based on these findings, I hypothesize that transplantation of young hematopoietic stem cells overcome extrinsic influences from the aged microenvironment to reverse the age-associated phenotype of the hematopoietic system. This hypothesis will be tested by pursuing the following three aims: 1) to establish age-associated phenotype in young stem cells within the aged niche; 2) to determine the overall lifespan of older recipient mice transplanted with young donor stem cells; and 3) to determine the effects of young stem cell transplantation on age-related frailty. The first aim examines the extent of which extrinsic factors from the aged microenvironment influences well established age-associated phenotypes of HSCs. This will be accomplished by investigating these phenotypes in young HSCs after prolonged exposure to the aged microenvironment *via* our non-cytotoxic HSCT method. The second aim includes a longevity study to distinguish the health-associated benefits of transplanting young HSCs into an older recipient. Since young HSCs exhibit young phenotypic features, they would represent rejuvenated HSCs as the “ideal” model to examine rejuvenation of the hematopoietic system. The third aim will investigate if young donor HSCs prevent or delay the onset of frailty by reducing chronic inflammation. The proposed research addresses essential questions before this technology can be translated into a clinical setting. This approach is particularly attractive because, in addition to eliminating the need for irradiation or chemotherapy, each of the essential reagents have previously been approved for human use through the FDA. Thus, the proposed research has the potential to improve current transplantation regimes by replacing traditional HSCT methods with our novel approach and may lead to the development of the first cell-based anti-aging therapy.

*Correspondence to: Michael Guderyon, University of Texas Health Science Center at San Antonio. Email: Guderyon@uthscsa.edu

## mTOR promotes BBB breakdown and dysregulation of tight junction proteins in an Alzheimer’s disease model

Stephen F. Hernandez*, Candice E. Van Skike, Dick DeRosa, Veronica Galvan

Barshop Institute, UT Health San Antonio, Texas

Cerebral amyloid angiopathy (CAA) is characterized by fibrillar amyloid β (Aβ) association with cerebrovasculature, which leads to impaired brain vascular function, and is present in 87% of people with Alzheimer’s disease (AD). Previously, it has been shown that inhibition of mTOR by rapamycin prevented vascular leakage in 18-19 month old Tg2576 mice – a mouse model that mimics AD-associated CAA. This finding suggests that mTOR plays a role in regulating the integrity and permeability of the blood brain barrier (BBB). To further expand on this study, the abundance of tight junction proteins, zonula occludens 1 (ZO-1), occludin, and claudin -5 were examined using immunofluorescent confocal microscopy on frozen brain tissue sections of the same Tg2576 mice from the previous study. Together, these studies demonstrate that attenuation of mTOR by rapamycin preserves BBB integrity by decreasing the amount of vascular Aβ accumulation, which also showed a reduced likelihood of cerebral micro hemorrhages and an increase in tight junction protein abundance in our transgenic Tg2576 mice. Therefore, these data suggest that mTOR is a key component in vascular Aβ accumulation, BBB breakdown, vascular dysfunction, and BBB permeability in the Tg2576 mice. Thus, the data suggests that using mTOR inhibitors such as rapamycin – an FDA approved drug, may be therapeutic in the pathogenesis of AD and other dementias with related cerebrovascular dysfunction.

*Correspondence to: Stephen F. Hernandez, Barshop Institute, UTHCSA, TX. Email: hernandezs23@uthscsa.edu

## Prion-like properties of tau oligomers trigger brain vascular endothelial cell dysfunction

Stacy Hussong^1,2,*^, Angela B. Olson^1,2^, Matthew Hart^2,3^, Rakez Kayed^4^, Veronica Galvan^1,2^

^1^Department of Cellular & Integrative Physiology, ^2^Barshop Institute for Longevity and Aging Studies, ^3^Deparment of Molecular Medicine at the University of Texas Health Science Center at San Antonio. ^4^Department of Neurology at University of Texas Medical Branch at Galveston.

Extracellular amyloid plaques and intracellular neurofibrillary tau tangles are the two major hallmarks of Alzheimer’s disease. Tau protein is involved in the stabilization of the microtubule cytoskeleton in neurons and other cell types. When tau becomes hyperphosphorylated, it disassociates from microtubules and forms tau oligomers which can then further aggregate into neurofibrillary tangles. It has been proposed that hyperphosphorylated, misfolded tau species propagate between neurons in a prion-like fashion, causing the aggregation of native tau in the target cell. Here we show that tau oligomers can propagate to endothelial cells. Our *in vitro* in primary human brain microvascular endothelial cells (HBECs) showed that tau oligomers are taken up by HBECs, and cause increased endogenous tau phosphorylation, followed by a decrease in microtubule density and stability. Activation of endothelial nitric oxide synthase (eNOS), a critical event in the pathway of NO release by vascular endothelial cells, is partly regulated by localization and trafficking to the cell surface via the microtubule cytoskeleton. Consistent with the observed decrease in microtubule cytoskeleton density and stability, we showed that eNOS activation is decreased in HBECs treated with oligomeric tau. Furthermore, our data shows that oligomeric can induce endothelial cell senescence. Expressions of several markers of senescence were increased after exposure to oligomeric tau including p16, p21, and IL-6. Together these data suggest that oligomeric tau can impair endothelial cell function as measured by the activation of eNOS and endothelial senescence.

Taken together with prior studies showing that misfolded tau can propagate in a prion-like fashion between neurons, our data suggest that tau oligomers can be specifically internalized by brain vascular endothelial cells and cause misfolding of endogenous native tau protein, destabilizing microtubules and decreasing the activation of eNOS. Because brain vascular dysfunction mechanistically contributes to the etiology of Alzheimer’s disease, cerebrovascular oligomeric tau may be a novel target for therapeutic development in Alzheimer’s disease.

*Correspondence to: Stacy Hussong, Barshop Institute, UTHCSA, TX. Email: hussong@uthscsa.edu

## Increased hepatic steatosis and acylcarnitine levels in mice treated with formoterol, a beta2-adrenergic receptor agonist

Xiaoli Gao^1^, Yun Shi^2,3^, Dana M. Molleur^1^, Susan T. Weintraub^1^, Amrita Kamat^2,3,4*^

^1^Biochemistry and Structural Biology, ^2^Barshop Institute for Longevity and Aging Studies, ^3^University of Texas Health San Antonio and GRECC, ^4^South Texas Veterans Health Care System, San Antonio, TX

Hepatic steatosis, a salient feature of non-alcoholic fatty liver disease, increases with age. Circulating catecholamine levels also increase with age and act via beta adrenergic receptors (β-ARs) to regulate lipid metabolism. In the current study, we investigated the relationship between augmented signaling via the β2-AR subtype and hepatic steatosis. We used a lipidomic approach to examine the influence of the administration of formoterol, a β2-AR agonist, on hepatic lipid profile changes and the underlying mechanisms involved in β2-AR agonist-induced hepatic steatosis. Among the lipids of particular interest for this study were acylcarnitines due to their critical involvement in dyslipidemia and related signaling pathways. Fifteen acylcarnitines were identified and quantified in the liver samples and 16 in plasma. In the liver, 11 acylcarnitines were significantly higher in formoterol-treated samples compared to the controls. As measured in a separate set of extracts, liver triglycerides were markedly elevated (>8 fold) after formoterol treatment. Based on the high levels of hepatic triglyceride accumulation in the treated animals, we hypothesized that fatty acid oxidation would be reduced in the treatment group. However, the increased levels of hepatic acylcarnitines suggest that formoterol treatment may increase fatty acid flux, resulting in an imbalance between uptake/synthesis of fatty acids in the liver and their removal by beta-oxidation. Currently, we are investigating β2-AR-mediated mechanisms that may contribute to the increased level of fatty acids in the liver, including elevated fatty acid uptake and *de novo* lipogenesis. We are also determining if *de novo* lipogenesis in the liver is occurring in concert with alterations in levels of enzymes associated with the beta-oxidation of fatty acids.

*Correspondence to: Amrita Kamat, Barshop Institute, UTHCSA, TX. Email: kamat@uthscsa.edu

## The role of microtubule cytoskeleton in regulating nuclear architecture and gene expression during aging

Su-Hyuk Ko*, Aiping Xu and Lizhen Chen

Barshop Institute for Longevity and Aging Studies, Department of Cell Systems and

Anatomy, University of Texas Health Science Center San Antonio, TX

Microtubules (MTs) are essential cytoskeleton involved in cell division, shaping the cell and intracellular transport. Mutations in tubulin genes or microtubule-associated proteins have been reported to affect neuronal integrity and function during aging. We have recently found that mutations in microtubule regulating genes can modulate C. elegans lifespan. Our data suggest that the effect of microtubule stability on lifespan is dependent on DAF-16, the sole C. elegans FOXO homolog that function as a transcription factor in the insulin signal pathway. Among the previously identified direct targets of DAF-16, unc-84 encodes an inner nuclear membrane protein that connects microtubule cytoskeleton and nuclear envelope. Interestingly, we found that the lifespan extension phenotype induced by microtubule stabilization could be partially blocked by loss of UNC-84, while loss of UNC-84 alone does not affect lifespan. We also found that mutations in microtubule regulating genes can affect neuronal nuclear size and shape. Together these data suggest that the effect of microtubules on aging might be partially through regulating nuclear architecture and gene expression. We are currently examining the effect of microtubule disruption on nuclear organization and gene expression by confocal imaging, ATAC-seq and RNA-seq.

*Correspondence to: Su-Hyuk Ko, Barshop Institute, UTHSCSA, TX. Email: KoSH@uthscsa.edu

## Creating a *Drosophila melanogaster* model of prion-like tau protein spread

Simon A. Levy*, Bess Frost

The University of Texas Health Science Center at San Antonio

Tauopathies are a class of neurodegenerative diseases characterized by the accumulation of fibrillar tau protein aggregates. In Alzheimer’s disease, tau pathology propagates hierarchically over time and appears to spread along brain circuitry. Recently, mounting evidence has surfaced to support the hypothesis of a prion-like mechanism for tau spread. Tau can spread between cells and form stably transmitted strains *in vitro* and *in vivo*; however, the mechanisms underlying this have not been well characterized. We propose to create a *Drosophila melanogaster* model of the prion-like spread of pathogenic tau based on the *Drosophila* olfactory circuitry, part of which consists of mushroom body output neurons (MBONs) projecting to five target regions. We will use a two-pronged approach to model the key features of prion-like protein propagation: 1) spreading of tau to other cells and 2) a capacity to recruit native tau to seed aggregation. A split-Gal4 system will direct expression of a seed-competent tau mutant to a subset of MBONs. The spread of tau will then be assessed by immunofluorescence. To detect the capacity of tau to spread to synaptically connected regions and seed aggregation of native tau protein, the same split-Gal4 system will be used to express seed competent tau fused to the N terminal half of luciferase (Luc^N^) in MBONs, while a LexA system will drive tau^WT^ fused to the C terminal half of luciferase (Luc^C^) expression in target regions. Luc^N^-tau spreading and recruiting the local Luc^C^-tau to seed aggregation will reconstitute functional luciferase and emit bioluminescence. A *Drosophila* model of tau propagation will allow us to investigate the mechanisms controlling this phenomenon, with the long-term goal of developing novel treatment strategies. In this context, a bioluminescence-based model of tau propagation is well suited for screening of genetic and pharmacological interventions that interfere with putative transmission mechanisms.

*Correspondence to: Simon A. Levy, UTHSCSA, TX. Email: levys@livemail.uthscsa.edu

## Testing the aldehyde hypothesis of Parkinson’s disease: the role of α-synuclein

Anthony Martinez*, Vanessa Martinez, Elizabeth Fernandez, Randy Strong

Barshop Institute for Longevity and Aging Studies, UT Health Science Center, San Antonio

Parkinson’s disease (PD) is the second most common neurodegenerative disorder. The cause of PD remains unclear, but many studies implicate oxidative stress. Biogenic aldehydes are among the most destructive sources of oxidative stress. The aldehydes, 4hydroxynonenal (4HNE), the end-product of lipid peroxidation, and 3,4dihydroxyphenylacetaldehyde (DOPAL), an intermediate in dopamine metabolism, have been shown to be elevated in brains of PD patients. Other studies link α-synuclein (αSyn) to PD. Gene mutations in αSyn in mouse models are associated with behavioral and pathological manifestations of Parkinson’s disease, providing evidence that αSyn plays a role in the neuropathology of this disease. Biogenic aldehydes have been reported to promote the formation of neurotoxic αSyn oligomers, while blocking the formation of less toxic fibrils *in vitro*. Our working hypothesis is that αSyn is mechanistically related to the behavioral, neuropathological and neurochemical manifestations of PD resulting from elevated biogenic aldehydes. To test this hypothesis we crossed mice overexpressing human wildtype αSyn under the control of the Thy1 promoter (Thy) with mice homozygous null for genes coding for the only two aldehyde dehydrogenase isozymes known to be expressed in nigrostriatal dopamine neurons, Aldh1a1 and Aldh2 (DKO). The result is a triple transgenic line (TTG) with overexpression of αSyn in the presence of elevated levels of biogenic aldehydes. In initial studies, the TTG genotype was associated with exacerbated motor deficits on the accelerate rotarod, pole test, adhesive removal test, and impaired gait performance, as compared to wild-type mice and their genotype controls. L-DOPA treatment is used clinically to diagnose PD. Therefore, we tested the effects of L-DOPA on motor performance in our models of PD. The results will be discussed.

*Correspondence to: Anthony Martinez, Barshop Institute, UTHSCSA, TX. Email: martinezp3@uthscsa.edu

## Phosphatidylglycerol remodeling regulates ER linked mitochondrial fission and programmed cell death

Kennedy S. Mdaki^*1^, Jia Nie^1^, Jun Zhang^1^, John-Paul Andersen^1^, Haoran Sun^2^, and Yuguang Shi^1,2^

^1^Barshop Institute for Longevity and Aging Studies, Department of Pharmacology, University of Texas Health Science Center at San Antonio, TX, ^2^Key Laboratory of Human Functional Genomics of Jiangsu Province, Jiangsu Diabetes Center, Nanjing Medical University, Nanjing, People’s Republic of China

Mitochondria undergo excessive fission during the manifestation of programmed cell death but the underlying cause remains unclear. Accumulating evidence shows that phosphatidylglycerol (PG), a phospholipid known for its role in cardiolipin synthesis, increases dramatically prior to cell death. However, how PG may be involved in programmed cell death is unknown. Given that cardiolipin detachment from mitochondria is one of the commitment steps in programmed cell death and PG levels spike during programmed cell death, we decided to investigate a possible mechanism by which PG may regulate programmed cell death. We found that newly remodeled PG induces programmed cell death via a mechanism that links mitochondrial depolarization, excessive fission, calcium overload, with plasma membrane permeabilization. We show that PG induced programmed cell death can be blunted by DIDS, necrostatin-1, and cycloheximide as well as exacerbated by pro-apoptotic TNFα, LCL161, and ABT-737. Furthermore, we report an unexpected role of PG on induction of mitochondrial fragmentation, of which we found to occur independent of Drp1. Taken together, our work puts forward a novel mechanism by which PG remodeling may play a fundamental role in the regulation of the different forms of programmed cell death.

*Correspondence to: Kennedy S. Mdaki, Barshop Institute, UTHSCSA, TX. Email: mdaki@uthscsa.edu

## Can changes to the neuronal proteasome system delay age related declines?

Erin Munkácsy Ph.D. ^*1^, Andrew M. Pickering Ph.D.^1^

^1^ Barshop institute of Aging Studies and Longevity, UT Health Science Center San Antonio, San Antonio, TX 78245

Disrupted proteostasis, increased damage and aggregated proteins are hallmarks of aging. A concomitant age-associated decline in proteasome activity has been reported across phyla and in multiple tissues in both mice and humans. The Prosβ5 proteasome subunit has been reported by our group and others to be rate limiting to proteasome synthesis. We have found that overexpressing the Prosβ5 subunit increases activity and function of both the 20S and 26S proteasome systems. When Prosβ5 expression is directed to the nervous system we see an extension in median and maximum lifespan. We also observe an amelioration of age-related cognitive deficits. Intriguingly, our data suggests that this effect may be non-cell-autonomous; boosting neuronal proteasome appears to drive whole organismal changes in the proteasome system. We are now attempting to determine whether the lifespan extension and delay in cognitive deficits are driven by changes to the 20S or 26S proteasome system and to develop a mechanistic understanding of the how neuronal proteasome can drive whole organism proteasome induction.

*Correspondence to: Erin Munkácsy, Barshop Institute, UTHSCSA, TX. Email: Munkacsy@uthscsa.edu

## SAD-A kinase function in pancreatic β-cells

Jia Nie^*1^, Yuguang Shi^1^, Nicolas Musi^1^

Barshop Institute for Longevity and Aging Studies, University of Texas Health Science Center San Antonio, TX

Type 2 diabetes mellitus (T2DM) is characterized by insulin resistance and β-cell failure, which leads to the depletion of islet β-cell mass. Understanding the molecular triggers of postnatal pancreatic β-cell proliferation may facilitate the development of regenerative therapies for diabetes. The key players during β-cell expansion are tuberous sclerosis complex 1 and 2 (TSC1/2) and the mechanistic target of rapamycin complex 1 (mTORC1) cascade, which integrate growth factors and nutrients. However, how TSC/mTOR signaling regulates β-cell proliferation is not completely understood. The Synapses of Amphids Defective protein kinase A (SAD-A), also called brain serine/threonine kinase (BRSK2), belongs to the AMP-activated protein kinase-related kinase (AMPK) family; it is primarily expressed in the brain and pancreas, and little in the testis. SAD-A kinase is the first tissue-specific mediator of glucose response in islet β-cells, and it controls islet β-cell size by mediating mTORC1 signaling [1-3]. The preliminary studies showed overexpression of SAD-A increased β-cell proliferation from both cultured islets and the MIN6 β-cell line, whereas knockout SAD-A increased β-cell apoptosis during multiple low-dose streptozotocin treatments in pancreas-specific knockout SAD-A mice and controls. Furthermore, the target deletion of SAD-A in the pancreas would inhibit islet proliferation response to a high-fat diet by decreasing the bromodeoxyuridine (BrdU) incorporation rate and cyclin D2 mRNA was elevated in SADA knockout mice. These results led to hypothesize that SAD-A controls postnatal pancreatic β-cell growth by triggering the entry of quiescent β-cells into the cell division cycle.

*Correspondence to: Jia Nie, Barshop Institute, UTHSCSA, TX. Email: niej@uthscsa.edu

## mTOR regulates PICALM in brain vascular cells

Angela Olson, Stacy Hussong*, Jordan Jarhling,, Veronica Galvan

University of Texas Health Science Center, San Antonio, TX

Advanced age is the greatest known risk factor for the development of Alzheimer’s disease (AD). A hallmark of AD is the accumulation of amyloid beta (Aβ) in brain, the majority of which is cleared from brain into the bloodstream across the blood-brain barrier (BBB) via transcytosis mediated by the low-density lipoprotein receptor-related protein 1 (LRP1). Several recent genome-wide association studies (GWAS) have implicated the allele *rs3851179*^A^ near the phosphatidylinositol-binding clathrin assembly protein (PICALM) gene as a protective against the development of AD. This allele is associated with an increase in mRNA expression of PICALM, which is abundant in brain endothelial cells (EC). PICALM binds to LRP1 to initiate transcytosis by recruiting clathrin to form clathrin-coated vesicles, ultimately removing toxic Aβ from the brain.

In the present study, we tested the hypothesis that mTOR may contribute to increased Aβ brain levels by reducing LRP1-mediated transport across BBB via a reduction in PICALM. To test this hypothesis, we measured protein levels and mRNA expression of PICALM and LRP1 in cultured endothelial cells and in brain microvasculature of transgenic AD mice that were treated with control or with the mTOR inhibitor rapamycin. While mTOR attenuation did not affect PICALM nor LRP1 mRNA expression levels, our studies demonstrated significant increases in PICALM protein levels in rapamycintreated cultured EC and in microvasculature of rapamycin-treated AD mice, suggesting that mTOR attenuation may increase PICALM *in vitro* and *in vivo*. The observed increases in PICALM levels as a result of rapamycin treatment were recapitulated by knock-down of Raptor, the obligatory companion of mTOR complex 1. Our data suggest that mTOR-mediated reduction of PICALM may contribute to increased brain Aβ levels in mice modeling AD, thus revealing a novel mechanism by which mTOR attenuation may reduce brain Aβ levels and significantly delay or stop the progression of AD.

*Correspondence to: Stacy Hussong, Barshop Institute, UTHSCSA, TX. Email: hussong@uthscsa.edu

## 17α-estradiol inhibits oxidative stress and Aβ release in two *in vitro* models of Aβ production

J.N. O’Neil^*1^, G. Xu^1^, P.R. Mouton^2^, Y. Tizabi^1^, K.F. Manaye^1^

^1^Howard University College of Medicine, Washington, DC; ^2^University of South Florida College of Medicine, Tampa, FL;

Alzheimer’s disease (AD), a progressive age-related dementia, diagnosed at autopsy by the presence of β-amyloid (Aβ) plaques, neurofibrillary tangles, and severe neuron loss. Although the etiology of AD is currently unknown, oxidative stress from the production of reactive oxygen species (ROS) causes neural apoptosis and stimulates Aβ production. In this study, we tested the hypothesis that using 17α-estradiol (17αE2), the non-feminizing structural analog of 17βestradiol (17βE2), might protect against apoptosis and Aβ deposition in two transgenic neuroblastoma mouse cell lines associated with Aβ production. The N2a-APP695 cell line expresses the Swedish mutation of amyloid precursor protein, APP695; the N2a-APP695/PS1DE9 cell line coexpresses PS1DE9, a presenilin mutation associated with a deletion at delta9 exon. Cell lines were pretreated overnight with either 17αE2 or vehicle followed by exposure to ROS-generators hydrogen peroxide (H2O2) or glucose oxidase (G/GOx) to induce oxidative stress. The ability of 17αE2 to protect cells against apoptosis and mutant Aβ production was assessed by evaluating DHE and caspase-3 expression, Aβ1-40/42 release, and number of apoptotic cells, as determined by immunofluorescence, Western blotting, and flow cytometry. Results revealed that in cells exposed to H2O2 or G/GOx, 17αE2 treatment reduced the number of cells expressing DHE and caspase-3 significantly increased cell survival. Furthermore, pretreatment of N2a-APP695/PS1DE9 cells with 17αE2 reduced extracellular levels of Aβ in a dose-dependent manner, with no change in the intracellular levels of Aβ. Thus, 17αE2 appears to provide a potentially efficacious strategy that may slow the progression of AD, without the adverse side effects associated with 17βE2.

This research was supported by grants NIH/NIA R03AG0449288 and NIH/NIA R25AG0478433. The authors would like to thank Dr. Huaxi Xu of Burnham Institute for Medical Research for providing these cell lines.

*Correspondence to: J. N. O’Neil, Howard University College of Medicine, Washington DC. Email: jahn.oneil@howard.edu

## Tau protein aggregation induces cellular senescence in the brain

Joseph M. Valentine^1^, Kathryn R. Sickora^1^, Cody S. Thompson^1^, Ashley Zapata^1^, Nicolas Musi^1,2^, Miranda E. Orr^*1,2,3^

^1^Barshop Institute for Longevity and Aging Studies, University of Texas Health Science Center at San Antonio, 15355 Lambda Drive, San Antonio, TX, 78245, USA. ^2^San Antonio Geriatric Research, Education and Clinical Center, South Texas Veterans Health Care System, 7400 Merton Minter, San Antonio, TX, 78229, USA. ^3^Department of Pharmacology, Center for Biomedical Neuroscience, University of Texas Health Science Center San Antonio, 7703 Floyd Curl Drive, San Antonio, TX, 78229, USA.

Tau protein accumulation is the most common pathology among brain diseases, including Alzheimer’s disease (AD), progressive supranuclear palsy (PSP), chronic traumatic encephalopathy (CTE) and over twenty others^1^. Mechanisms mediating tau toxicity are poorly understood. Tau-containing neurofibrillary tangle (NFT) accumulation is the closest correlate with cognitive decline and cell loss, yet NFT-containing neurons do not die suggesting the involvement of secondary mechanisms^2,3^. To gain insight into NFT-mediated toxicity, we evaluated gene expression patterns of NFT-containing neurons microdissected from AD patient brains^4^. We find that they display a gene expression profile consistent with cellular senescence. This complex stress response induces permanent cell cycle arrest in dividing cells, apoptosis resistance, cellular remodeling and metabolic dysfunction^5^. Senescent cells induce chronic degeneration of surrounding tissue through the secretion of pro-inflammatory, pro-apoptotic molecules termed the senescence-associated secretory phenotype (SASP)^6^. Using transgenic mouse models of tau-associated pathogenesis we find that NFTs induce a senescence-like phenotype in the brain including DNA damage, karyomegaly, mitochondrial dysfunction and SASP. *Cdkn2a* transcript level, a hallmark measure of senescence, directly correlates with brain atrophy and NFT load. We find this relationship extends to humans with PSP suggesting a phenomenon common to tau toxicity. Moreover, like in the human population where women have increased risk of developing AD, we find female mice to be more greatly affected by cellular senescence to suggest its role in sex-dependent pathophysiology. In summary, we identify cellular senescence, the quintessence of latent tissue degeneration, as an anti-apoptotic mechanism of NFT-containing neurons in AD. Our results suggest that NFTs formed in early pathogenic stages may contribute to neurodegeneration through senescence-like mechanisms by altering the bioenergetic state of the brain and inducing the release of toxic SASP. Therapeutically targeting senescent cells, or their secondary pro-apoptotic byproducts, may interrupt perpetual chronic neurodegeneration common to AD and >20 additional tau-associated diseases, and is an area of our ongoing research.

*Correspondence to: Miranda Orr, Barshop Institute, UTHSCSA, TX. Email: orrm3@uthscsa.edu

## HSP25 overexpression extends lifespan through protective aggregation in an HSF1dependent manner

Karl A. Rodriguez^*1,2^, Courtney Carroll^1^, Tamara Fraker^1,2^, Chelbee Farnen^1,2^, Aliyah

Encarnacion^1^, Victoria Nguyen^3^, Maruf Khan^4^, Rochelle Buffenstein^5,6^, and Alfred Fisher^1,4,7,8^

^1^Sam and Ann Barshop Institute for Longevity and Aging Studies, University of TexasHealth Science Center San Antonio (UTHSCSA), San Antonio, TX 78229; ^2^Department of Cell Systems and Anatomy, UTHSCSA; ^3^Howard University, Washington, D.C.; ^4^Department of Medicine, UTHSCSA; ^5^Department of Pharmacology, UTHSCSA; ^6^Calico, 1170 Veterans Blvd, San Francisco, CA 94080; ^7^Center for Healthy Aging, UTHSCSA; ^8^GRECC, South Texas VA Healthcare System, San Antonio, TX 78229

Long-lived animals show resistance to a broad spectrum of environmental stressors. Our previous work indicated that this is attributed in part to altered changes in molecular chaperone levels that influence the transport and disposal of damaged proteins. Particularly striking were the high levels of the small molecular weight chaperone heat-shock chaperone 25 kDa (HSP25) and its putative transcription factor heat shock factor-1 (HSF-1). These showed as significant correlation with rodent longevity and were 5-fold higher our longest-lived species compared to the shortest. This suggests that HSP25 may play a key role in age-related maintenance of protein homeostasis in long-lived animals. To examine if this correlation was more causal we constructed transgenic *C. elegans* worms which ubiquitously overexpress HSP25 fused to GFP. Worms overexpressing this construct show resistance to heat stress (20% increase in mean survival) and have a 25% extension of lifespan. This lifespan extension depended on the expression of Hsf-1 but not Daf16 as determined by RNAi experiments. Generally, an increase life span and heat resistance corresponds with decreased aggregation. However, when we crossed our longer-lived, heat-resistant worms with worms containing a repeat of 44 glutamines (PolyQ) targeted to the worm intestine (Q44::YFP) we surprisingly observed that the HSP25 transgenic worm had a higher percentage of animals showing aggregation and more aggregates per worm than the control-crossed worm. Yet the lifespan of the HSP25 transgenic animals was still longer. This could suggest a role for this protein in promoting controlled aggregation which then removes unstable and/or damaged proteins by packaging them into insoluble aggregates. In theory, these now sequestered, aggregated proteins might be removed by autophagy or other protein degradation pathways which we are currently testing. We are also testing whether this phenomenon is specific to PolyQ aggregates or is a general process by examining HSP25 overexpression in worms containing control and mutant human tau. The sequestration of these aggregate-prone structures by HSP25 and other sHSPs could be a protective mechanism within the cell.

*Correspondence to: Karl Rodriguez, Barshop Institute, UTHSCSA, TX. Email: rodriguezk@uthscsa.edu

## Serum proteins mediate gender’s association with dementia

Royall DR^*1-4^, Al-Rubaye S^1^, Palmer RF^3^

Departments of Psychiatry^1^, Medicine^2^, Family & Community Medicine^3^, The University of Texas Health Science Center and the South Texas Veterans Health Administration Geriatric Research Education and Clinical Center (GRECC)^4^

Background: Alzheimer’s Disease (AD) is diagnosed in women more often than in men, and female gender is a risk factor for incident dementia and MCI conversion. The latent variable “δ” (for “dementia”) appears to be uniquely responsible for the dementing aspects of cognitive impairment. Age, depression, the apolipoprotein E (APOE) e4 allele and gender are independently associated with δ. Methods: In this analysis, we explore serum proteins as potential mediators of gender’s specific association with δ in a large, ethnically diverse, longitudinal cohort, the Texas Alzheimer’s Research and Care Consortium (TARCC). N = 3381 participants were followed prospectively. N = 115 serum proteins were measured at baseline and screened as mediators of gender’s prospective association with year 2 δ scores. Models were adjusted for age, APOE, education, ethnicity, depression ratings, homocysteine and hemoglobin A1c. Significance was Bonferroni corrected to p <0.001. Significant effects were replicated in random 50% subsets of TARCC’s sample. Results: 17 proteins were found to be full or partial mediators of gender’s effect on δ. Several sex hormones (i.e., FSH, LH, and testosterone) strongly moderated gender’s effect. Prostatic acid phosphatase (PAP) fully mediated gender’s effect. Other sex hormones (i.e., progesterone and prolactin) were not associated with gender in this (post-menopausal) sample. The remaining 13 proteins had partial mediation effects. Most were weak, but Thyroxine Binding Globulin (TGB) explained a sizable fraction of Gender’s effect (75.5%). Gender’s mediators did not overlap substantially with those we have previously associated with age, or depression. However, C-reactive protein (CRP) mediates both Gender and APOE’s effects, and may explain Gender’s published interaction with APOE’s dementia risk. Similarly, BDNF’s association with dementia is reported to be gender specific, consistent with our findings. Conclusions: This analysis identifies serum protein targets for the modulation of gender’s specific effect on dementia severity and AD conversion risk. The selected proteins may need to be associated with dementia in gender stratified, rather than gender adjusted analyses.

*Correspondence to: Donald Royall, Department of Psychiatry, Medicine, Family & Community Medicine, UTHSCSA, TX. Email: royall@uthscsa.edu

## Metabolic effects of donor sex persist *ex vivo* in primary cell cultures

Carlos Galeano, Yuhong Liu and Adam Salmon*

The Sam and Ann Barshop Institute for Longevity and Aging Studies, UT Health San Antonio and Geriatric Research, Education and Clinical Center, South Texas Veterans Health Care System, San Antonio TX

While there are numerous significant sex differences in physiological characteristics, the basis for these sexual dimorphisms is not completely evident. Sex hormones certainly play a significant role, though evidence is growing that basic genetic differences may also differentiate males from females. To test this idea, we generated lines of primary fibroblasts from young adult HET3 mice that were expanded under standard culturing conditions. That is, native cells derived from mice were exposed to normal *in vivo* environments, including sex hormones, through 12 weeks of age but after isolation were grown in media containing identical culture media and serum. These cell lines retained metabolic properties dependent on their genetic sex/sex of donor even after more than 3 weeks in culture. Fibroblasts from male HET3 mice display higher basal oxygen consumption rates (+27%), are less resilient to mitochondrial uncoupling due to FCCP (+42%), and devote more of their oxygen consumption to ATP generation (+27%) than do female-derived cells, suggesting an increased need for energy utilization in genetically male cell cultures. For each, the variability among male-derived cells in each of these measures was approximately two-fold that of female-derived cells. It is unclear whether sexual differences in cellular physiology level are due to previous exposure to sex-specific hormone concentrations *in vivo* or to fundamental differences at the cellular level (*i.e*., XX or XY genetics). Our future studies will address these questions as well as test whether these basic cellular sex differences might contribute to the robust survival advantage of women over men globally.

This work was supported by grants from NIA (R01-AG050797 and R01-AG057431).

*Correspondence to: Adam Salmon, Barshop Institute, UTHSCSA, TX. Email: salmona@uthscsa.edu

## Tau gene expression regulates sex-dependent differences in obesity, cellular senescence and Alzheimer’s disease pathogenesis in transgenic mice

Kathryn R. Sickora and Miranda E. Orr*

University of Texas Health Science Center at San Antonio, San Antonio TX, USA

Advanced age is the greatest risk factor for most chronic diseases, yet pathogenic mechanisms tend to be highly tissue specific and sex dependent. In brain, intraneuronal inclusions of tau protein are the most common pathology. Collectively referred to as “tauopathies,” these diseases encompass over 15 distinct disorders. Alzheimer’s disease (AD) is the most common tauopathy and disproportionately affects women, but through unknown mechanisms. To investigate tau-associated pathogenesis and sex-dependent effects, we use transgenic mice that model human AD tau pathogenesis, referred to here as tauNFT. Like humans, female tauNFT mice develop more severe pathology, neurodegeneration and cognitive decline than male mice. During tau pathogenesis, neurons accumulate hyperphosphorylated tau, soluble tau oligomers and eventually large insoluble neurofibrillary tangles (NFTs). NFTs are the closest histopathological correlate with neuron loss and cognitive decline in AD, but the neurons with NFTs do not die. Thus, the contribution of NFTs to AD pathophysiology remains unknown but suggests the contribution of alternative pathways and mechanisms. Using our mouse model of tauopathy, we recently found evidence of the involvement of cellular senescence, a cell stress pathway known to exert toxicity through non-cell autonomous mechanisms. Moreover, we found this cell stress pathway to be significantly higher in female than male mouse brains. However, when the endogenous mouse tau gene, *Mapt*, is genetically removed, female and male mice become equally affected indicating that removal of endogenous mouse tau may uniquely benefit female mice. We also find significant, and sex-dependent, changes in body weight with *Mapt* removal indicating its expression contributes to sexual dimorphic physiological traits. We are now using transgene suppression studies and stereotaxis to identify the brain region(s) responsible for these sex-specific effects. By using both male and female mice, the interplay among sex-specific differences among tau pathology, senescence, cognitive decline and obesity will be determined.

*Correspondence to: Miranda Orr, Barshop Institute, UTHSCSA, TX. Email: orrm3@uthscsa.edu

## Impact of sex on thyroid hormone protection against stroke

Sifuentes MM^*1^ and Lechleiter JD^2^

^1^Department of Pharmacology, ^2^Department of Cellular and Structural Anatomy University of Texas Health Science Center at San Antonio

Stroke is a leading cause of death and disability in the United States with a disproportionate impact on the elderly, who have a higher stroke risk and are less likely to recover from neurological and physical impairments. Research in our lab has identified acute treatment with thyroid hormone as a protective strategy against a photothrombotic model of permanent ischemia, but the influence of sex on cerebral photothrombosis and neuroprotection has not previously been reported. After collecting the brains of mice 24 hours post-stroke, we observed no significant difference between vehicle-treated male and female mice (t-test, p=0.39). However, we did detect an impact of sex on the thyroid hormone dose required to effectively reduce lesion size when treatment was delivered 30 minutes after stroke (two-way ANOVA, p<0.0001). Furthermore, we found that while the optimal dose for neuroprotection in female mice remains effective when administered up to 12 hours post-stroke, protection in males conferred by the 30-minute optimal dose rapidly declines as the time-to-treatment period increases (one-way ANOVA, p=0.02). Neuroprotection up to 12 hours post-stroke is restored by increasing T3 concentration, suggesting a time-dependent sensitivity to thyroid hormone treatment in male, but not female, mice (one-way ANOVA, p=0.01). In conclusion, our observations indicate an influence on sex on the thyroid hormone-mediated mechanism that mitigates brain damage following ischemic stroke.

*Correspondence to: Miranda Sifuentes, Barshop Institute, UTHSCSA, TX. Email: sifuentesmm@uthscsa.edu

## Loss of TMEM127 in liver protects against insulin resistance

Srikantan S^*1^, Deng Y^1^, Luo A^1^, Qin Y^1^, Cheng Z^1^, Gao Q^1^, Li Z^2^, Salmon A^3^, Dong L^2^, Reddick R^4^, Norton L^1^, Abdul-Ghani M^1^, Dahia PLM^1^.

^1^Depts of Medicine, ^2^Cellular & Systems Analytics, ^3^Molecular Medicine, ^4^Pathology, University of Texas Health Science Center at San Antonio, UTHSCSA, San Antonio, TX.

Inhibition of mTOR complex 1 (mTORC1) by rapamycin increases lifespan in mice and is clinically used to treat cancer. However, long term treatment of rapamycin also inhibits mTOR complex 2 (mTORC2) and promotes insulin resistance, limiting its use as an anti-aging or anti-cancer agent. TMEM127 is a lysosomal protein that impinges on mTORC1 through unknown mechanisms. To better characterize the TMEM127 gene we have generated mice with targeted deletion of *Tmem127* under a generic promoter. Chow diet-fed Tmem127 knockout (KO) mice have reduced body weight and fat mass, and are protected from age-related insulin resistance. When fed a high fat diet (HFD) the KO mice gained fat mass but were remarkably protected from hepatosteastosis-and diet-induced insulin resistance. mTORC2 complexes were more abundant in the liver of adult KO mice, both under chow and after HFD, in support of their higher insulin sensitivity. Chronic treatment with rapamycin caused down regulation of mTORC1 and mTORC2 signaling, but unlike WT, KO mice did not develop insulin resistance and their livers retained higher levels of mTORC2 complex. Liver-specific, but not adipose specific ablation of Tmem127 in mice resulted in increased insulin sensitivity suggesting a liver-specific role for Tmem127 in modulation of insulin sensitivity. We have generated HepG2 cells with CRISPR mediated knockdown of TMEM127 to investigate cell autonomous effects of TMEM127 in the liver, and to uncover the molecular mechanisms underlying these changes.

*Correspondence to: Subramanya Srikantan, Department of Medicine, UTHSCSA, TX. Email: subramanyasr@uthscsa.edu

## Pathogenic tau drives a toxic activation of transposable elements

Wenyan Sun^*1^, Ph.D., Hanie Samimi^2^, Miranda Orr^1^, Habil Zare^2^, Ph.D, Bess Frost^1^, Ph.D.

^1^Barshop Institute for Longevity and Aging Studies, Department of Cell Systems and Anatomy, UTHSA, San Antonio, Texas 78245, USA; ^2^Department of Computer Science, Texas State University, San Marcos, TX 78666, USA

Tauopathies, including Alzheimer’s disease, are age-related progressive neurodegenerative disorders that are pathologically defined by aggregates of tau protein in the brain. We have previously reported that tau-induced heterochromatin relaxation promotes neuronal death in tauopathies, and allows transcription of genes that are normally silenced by heterochromatin. We hypothesized that heterochromatin relaxation also causes transcription and mobilization of transposable elements in tauopathy. In support of this hypothesis, we find increased transposable element transcription and mobilization in a *Drosophila* model of tauopathy. Mechanistically, we find that transposable element activation is likely due to decreased levels of piRNAs, a class of small RNAs that target transposable element transcripts for silencing. Caloric restriction and treatment with an FDA-approved pharmacological inhibitor of reverse transcriptase, 3TC, decreases transposable element mobilization and neuronal death in tau transgenic *Drosophila*. We detect increased transposable element activation in a mouse model of tauopathy, and differentially expressed transposable elements in brain tissue from human Alzheimer’s disease and Progressive Supranuclear Palsy (a “primary” tauopathy) patients. Taken together, our findings suggest that pathological tau activates transposable elements, and that tau-induced activation of transposable elements is amenable to environmental and pharmacological inhibition. Our findings may lead to the development of predictive biomarkers and will guide therapeutic strategies.

## Acknowledgements

UTHSA Greehey Children’s Cancer Institute Genome Sequencing Facility

## Funding support

NINDS R00 and Owens Foundation grants awarded to B.F.

*Correspondence to: Wenyan Sun, Barshop Institute, UTHSCSA, TX. Email: sunw@uthscsa.edu

## Regulation of breast cancer progression by stromal DDR1

Xiujie Sun*, Kshama Gupta, Bogang Wu, Deyi Zhang^1^, Bin Yuan, Xiaowen Zhang, Huai-Chin Chiang, Chi Zhang, Yanfen Hu, Rong Li

Department of Molecular Medicine ^1^Department of Medicine, University of Texas Health Science Center at San Antonio, San Antonio, TX 78229, USA

Adipose stromal cells (ASC) constitute a significant component of breast stroma. Emerging evidence demonstrates a functional interaction between ASC and breast cancer (BC) cells. Here, we report that stromal discoidin domain receptor 1 (DDR1) is important for ASC-BC communication. Using a DDR1 knockout mouse model, we find that syngeneic mammary tumors grow more slowly in KO mice than wild-type littermate controls. In vitro, loss of DDR1 in stromal vascular fraction (SVF) impairs its ability to stimulate migration and invasion of tumor cells. Furthermore, we show that DDR1-dependent IL-6 production in SVF is responsible for the SVF effect on tumor cells. These results uncover a previously unrecognized role of stromal DDR1 in BC progression and a potential therapeutic target for BC treatment.

*Correspondence to: Xiujie Sun, Department of Molecular Medicine University of Texas Health Science Center at San Antonio, TX, Email: sunx4@uthscsa.edu

## Tau protein aggregation induces cellular senescence in the brain

Cody S. Thompson^*1^, Miranda E. Orr^1,2,3^, Joseph M. Valentine^1^, Kathryn R. Sickora^1^, Ashley Zapata^1^, Nicolas Musi^1,2^

^1^The Sam and Ann Barshop Institute for Longevity and Aging Studies, 15355 Lambda Drive, San Antonio, TX, 78245, USA. ^2^San Antonio Geriatric Research Education and Clinical Center, South Texas Veterans Health Care System, 7400 Merton Minter, San Antonio, TX, 78229, USA. ^3^Department of Pharmacology, Center for Biomedical Neuroscience, University of Texas Health Science Center San Antonio, 7703 Floyd Curl Drive, San Antonio, TX, 78229, USA.

Cellular senescence is a complex cell stress response involved with age-related decline in tissue function and structure. The senescence stress response is best studied in regenerative tissues; whether age-associated diseases affecting post-mitotic cells and tissues also involve cellular senescence remains unclear. To evaluate the relationship between cellular senescence and neurodegenerative disease, we use the rTg4510 mouse model of Alzheimer’s disease associated tau pathogenesis and neurodegeneration. We have observed several senescence biomarkers in the brain including p16^INK4A^ expression, senescence associated secretory phenotype (SASP) factors and senescence associated β-galactosidase activity, and karyomegaly in neurons. Moreover, mice possessing tau pathology expressed significantly higher levels of several senescence factors than controls, including p16^INK4A^, TNFα, IL-1β, Cxcl2, B2m, NFκB, and Tlr4, while also being karyomegalic. Collectively our data indicate that cellular senescence occurs in post-mitotic tissue, and that Alzheimer’s disease-associated tau pathology exacerbates the cell stress response. Our future studies are aimed at targeting cellular senescence as a new therapeutic approach to ameliorate Alzheimer’s disease pathogenesis and cognitive decline.

*Correspondence to: Cody S. Thompson, Barshop Institute, UTHSCSA, TX. Email: ThompsonCS@livemail.uthscsa.edu

## Methionine restriction: Mechanism and cell culture

Thyne KM^*1^, Liu Y^1^, Galeano CA^1^, Dorigatti JD^1^, Salmon AB^1^

^1^The Sam and Ann Barshop Institute for Aging and Longevity Studies 15355 Lambda Drive, San Antonio, Texas 78245

Methionine is an essential amino acid and is required for many biological processes such as methylation, protein translation, and cysteine production. Restriction of this single amino acid in the diet can provide numerous benefits in rodents including extension of lifespan, reduced body weight, and improved glucose metabolism. The mechanisms that regulate this process are at least partially distinct from the mechanisms by which calorie restriction mediates its effects. It has been proposed that sulfur metabolism and methionine processing are important in understanding the mechanisms of methionine restriction. To help elucidate these mechanisms, our goal is to develop a cell culture model to provide a new tool for studying methionine restriction in different cell types. Further, we address the central role of methionine biochemistry in these potential mechanisms. The sulfur atom in methionine is sensitive to oxidation, though this can be reduced by methionine sulfoxide reductases found in eukaryotic cells. Interestingly, mice lacking one of these reductases (*MsrA^−/-^*) do not respond to the metabolic effects of methionine restriction. How aging, or healthy aging, is impacted by the oxidation status of the methionine pool is not known, but these interactions are likely to change during the aging process and under dietary restriction. Moreover, these findings suggest functional expression of MsrA may be a requirement for the longevity benefits of methionine restriction.

*Correspondence to: Kevin Thyne, Barshop Institute, UTHSCSA, TX. Email: thyne@livemail.uthscsa.edu

## Sex differences in the human skeletal muscle transcriptome during aging and exercise training

Joseph M. Valentine^*1,2^; Sangeeta Ghosh^1,2^; Yousin Suh^5^; Brandon Milholland^5^; John Gelfond^2,4^; Nicolas Musi^1,2,3^

^1^University of Texas Health Science Center at San Antonio, ^2^Barshop Institute for Longevity and Aging Studies, ^3^San Antonio Geriatric Research, Education and Clinical Center, ^4^Department of Epidemiology & Biostatistics; University of Texas Health Science Center at San Antonio, ^5^Albert Einstein College of Medicine

Muscle mass and strength decline with age in both men and women, resulting in reduced physical function and quality of life. Endurance exercise training can mitigate and even reverse some of the large-scale changes in skeletal muscle structure and function. To date, few studies in humans have focused on sex differences in mechanisms responsible for sarcopenia and the adaptive response to exercise training during aging. To gain insight into the potential mechanisms driving sarcopenia and the adaptive response to endurance training in older men and women we conducted deep sequencing on *vastus lateralis* muscle obtained from healthy, younger (n=14, age=27±1 y) and older (n=14, age=71±1 y) adults before and after a 4 month endurance training program. We utilized this sequencing data to determine *miRNA*s and mRNA expression patterns in our study’s cohorts. In females, 23 miRNAs decreased with age and were reverted back to youthful levels with training. Conversely, in males only one *miRNA* showed this same expression pattern. Also, in males 4 *miRNA*s increased with age and were reverted back to youthful levels with training, whereas no *miRNA*s with these expression patterns were observed in females. Pathway analyses in older male (but not female) subjects revealed enrichment and activation of mTOR, FGF, and VEGF pathways in response to training. These pathways are important for the adaptive response to training by increasing protein synthesis/vascularization in muscle. In contrast, older female subjects showed enrichment and activation of inflammatory pathways such as NFκB, inflammasome, apoptosis, and JNK signaling, while these inflammatory pathways were not enriched in males. MicroRNAs miR-365-3p, miR-36073p had youthful signatures after training, in older men. These two miRNAs are predicted to target members of the PI3K/AKT signaling pathway and they were downregulated after training with concomitant upregulation of the mRNA transcripts (i.e. PIK3R3, PRKC1, AKT3, and SGK1) in our data set. Surprisingly, no *miRNA*/mRNA relationships of this kind were observed in older female subjects after training. Therefore, we conclude that the potential mechanisms responsible for sarcopenia and adaption to endurance training in older men and woman may vary between sexes. Furthermore in females, we find that *miRNA*s that changed with age and were reverted to youthful levels with training do not regulate mRNA expression changes induced by training in the older group. Yet, miRNAs with this expression patterning in older men are capable of regulating transcriptional changes including pathways important for insulin signaling and action.

*Correspondence to: Joseph Valentine, Barshop Institute, UTHCSA, TX. Email: valentinej@livemail.uthscsa.edu

## MTOR-dependent neuronal nitric oxide dysfunction underlies neurovascular coupling deficits in a model of Alzheimer’s disease

Candice E. Van Skike*, Stacy A. Hussong, Nick DeRosa, Stephen F. Hernandez, Megan E. Reyna, Veronica Galvan

Barshop Institute for Longevity and Aging Studies, Department of Cellular and Integrative Physiology -University of Texas Health San Antonio

Cerebrovascular dysfunction emerges prior to the onset of cognitive dysfunction in patients with Alzheimer’s disease (AD). Specifically, patients with AD exhibit deficits in neurovascular coupling (NVC, increased cerebral blood flow evoked by neuronal activation), which is recapitulated in various mouse models of AD. We have previously identified the mechanistic/mammalian target of rapamycin (mTOR) as a major driver of cerebrovascular dysfunction in AD. Therefore, the aims of the present study were to 1) establish the mTORdependent contribution to NVC deficits in AD mice, and 2) define the mechanisms of mTOR mediated neuronal nitric oxide synthase (nNOS) insensitivity using *in vivo* and *in vitro* techniques.

J20 mice at 6 and 12 months old had profound impairments in NVC, particularly in the nNOS-dependent component of NVC, which was preserved by attenuation of mTOR activity with rapamycin treatment for 8 weeks. Further, NVC deficits were present prior to cognitive impairments, as contextual memory was not significantly impaired in the 6MO mice. Our *in vitro* studies indicate that mTOR inhibition with rapamycin increases nNOS activation as measured by phosphorylation of Ser1177. Additionally, preliminary coimmunoprecipitation studies suggest that mTOR attenuation increases the binding of nNOS to its binding partners PSD95 and HSP90, which positively regulate nNOS. These studies establish that mTOR drives the loss of the nNOS component of NVC in an AD mouse model, possibly by inhibiting nNOS phosphorylation at Ser1177.

Funding: CVS: AARF-17-504221 and NIA-T32AG021890. SFH: R25NS080684. VG: 1I01-BX002211-01A2 VA Merit Award, Owens Foundation, SAMF, NIA/NIH-P30-AG013319-21 Nathan Shock Center of Excellence, and RL Bailey and daughter LK Bailey Alzheimer’s Fund.

*Correspondence to: Candice E. Van Skike, Barshop Institute, UTHCSA, TX. Email: vanskike@uthscsa.edu

## Immunosenescence drives systemic aging

Matt Yousefzadeh*, Rafael Flores, Erin Wade, Robert Brooks, Luise Angelini, Paul D. Robbins,

Laura J. Niedernhofer

Department of Molecular Medicine and the Center on Aging, The Scripps Research Institute – Florida, Jupiter, FL 33458

Aging is the primary risk factor for numerous chronic diseases and the impact of aging on the immune system contributes to this. The progressive decline of immune function that occurs with aging, termed immunosenescence, can negatively impact both lifespan and healthspan due to impaired response to immunologic stimuli. Immunosenescence is characterized by a shift in T cell populations, expression of senescence markers, and a decline in both cell-mediated and humoral immunity. These changes lessen the ability of the elderly to mount a productive immune response to pathogenic infection, increase the risk of autoimmune diseases and cancer, and diminish the response to vaccinations. It remains controversial whether immunosenescence is driven primarily by cell autonomous and/or non-autonomous events. We utilized a mouse model (*Vav-iCre^+/-^;Ercc1^−/fl^*) with increasing burden of endogenous DNA damage in immune cells to determine if this is sufficient to drive senescence and functional decline of the immune compartment. *Vav-iCre^+/-^;Ercc1^−/fl^* mice exhibited an early and progressive onset of lymphopenia and expansion of the memory T cell population, hallmarks of immunological aging. By 20 weeks, a diminished response to immunological stimuli was observed in comparison to controls and increased senescence marker expression in peripheral blood lymphocytes, a biomarker of aging, were increased in *Vav-iCre^+/-^;Ercc1^−/fl^* mice, similar to naturally aged wild-type mice. Interestingly, we also found increased senescence in the liver, kidney and brain, in addition to the spleen and bone marrow of *Vav-iCre^+/-^;Ercc1^−/fl^* mice, suggesting that immune cells are a key driver of secondary senescence and that immunosenescence helps promote systemic aging.

*Correspondence to: Matt Yousefzadeh, Scripps Research Institute, FL. Email: myousefz@scripps.edu

## The role of small RNAs in a Drosophila model of tauopathy

Gabrielle Zuniga*, Wenyan Sun, Maria Gamez, and Bess Frost

Sam and Ann Barshop Institute for Aging and Longevity Studies, University of Texas Health Science Center at San Antonio

The microtubule associated-protein tau is known to play a role in the pathogenesis of Alzheimer’s disease and other related disorders. While differential expression of some small RNAs has been previously correlated with Alzheimer’s disease in humans, it is currently unknown if alterations in small RNA expression are causal for the disease process. Recent small RNA sequencing data from our lab reveal differential expression of microRNAs (miRNAs), endogenous siRNAs (esiRNAs), and piwi-associated RNAs (piRNAs) in a *Drosophila* model of human tauopathy. miRNA have a well-established role in post-transcriptional regulation of gene expression, while piRNAs and esiRNAs are recently-described, highly conserved classes of small noncoding RNAs that post-transcriptionally silence transposable elements. Interestingly, the majority of differentially expressed miRNAs are increased in tau transgenic *Drosophila*, while the majority of differentially expressed esiRNAs and piRNAs are decreased in tau transgenic *Drosophila* compared to control. We are currently determining if differentially expressed small RNAs play a causal role in promoting neurotoxicity, and are investigating the mechanism underlying the expression differences among miRNAs, piRNAs, and esiRNAs. Our studies suggest that tau-induced dysregulation of small RNAs may mediate neuronal death in tauopathies.

*Correspondence to: Gabrielle Zuniga, Barshop Institute, UTHCSA, TX. Email: zunigag3@livemail.uthscsa.edu

